# Health beliefs on type 2 diabetes: a methodological research for development and psychometric evaluation of “DIABA” (Diabetes-related Instrument to Assess Beliefs of Adolescents) health beliefs on type 2 diabetes

**DOI:** 10.1186/s12887-023-04251-3

**Published:** 2023-08-25

**Authors:** Ameneh Pooresmaeil Dorosteh, Mohtasham Ghaffari, Sakineh Rakhshanderou, Yadollah Mehrabi

**Affiliations:** 1 School of Public Health and Safety, Shahid Beheshti University of Medical Sciences, Tabnak Ave., Daneshjou Blvd., Velenjak, Tehran, P.O. Box 19835-35511 Iran; 2 Department of Epidemiology, School of Public Health and Safety, Shahid Beheshti University of Medical Sciences, Tehran, Iran

**Keywords:** Adolescents, Development, Attitude, Methodological, Type 2 diabetes mellitus

## Abstract

**Background:**

Several reports have shown an increase in the number of type-2 diabetics among adolescents creating an extra burden for this age group. However, there is no instrument assessing adolescents’ attitude toward this disease. This study aims at designing a psychometric tool for assessing adolescents’ health beliefs regarding type-2 diabetes.

**Research design and methods:**

In this methodological research, 770 boy and girl adolescents (between 13 and 15) from Tehran participated through multistage sampling. The Inclusion criteria were: junior high school students, students’ willingness for participation and not suffering from type-1 or type-2 diabetes. The questionnaire was designed by extensive literature review and the related existing questionnaires, as well as considering the research team’s comments. The validity of the questionnaire was determined through face and content validity. The construct validity was determined through exploratory and confirmatory factor analysis. Reliability was measured via internal consistency coefficient (ICC) and internal consistency reliability was measured by Cronbach Alpha. SPSS 16 and EQS6.1 were used for data analysis.

**Results:**

The pool of questions had 57 items, and by removing similar (23 questions) or inappropriate sentences (8 questions), a draft questionnaire with 26 questions was designed. No items were removed in the face validity phase. Based on the results of CVR and CVI, six items and 4 items in the exploratory factor analysis were removed. Finally, a questionnaire with 16 items in 4 dimensions of perceived self-efficacy, behavioral beliefs, perceived susceptibility and perceived severity was obtained. The results of confirmatory factor analysis confirmed the model. The internal consistency coefficient was confirmed measuring Cronbach Alpha at 0.78 and ICC = 0.73.

**Conclusion:**

The questionnaire designed can be employed as a reliable and valid instrument to assess the psychological perceptions and health beliefs of adolescents with respect to type-2 diabetes.

## Background

Type-2 diabetes is recognized to be one of the four main non-infectious diseases troubling the public health system in both developed and developing countries [1] and constitutes 90% of all cases of diabetes [2]. During the last three decades, the global number of diabetics has doubled [3]. In 2019, 463 million adults between the ages of 20 and 79 were suffering from type-2 diabetes. This figure is estimated to rise to 700 million by 2045 [4]. Also, the past 20 years have seen the rate of type-2 diabetes to multiply among children and adolescents in the US as well as other parts of the world [5] and has, only recently, become recognized as a pediatric disease in clinical settings [6]. Based on the results of the Tehran’s Sugar and Lipid study, the prevalence of type-2 diabetes is 1% among Iranian adolescents of 10 to 19 years of age [7].

Type-2 diabetes can contribute to kidney complications, blindness, amputation of extremities, cardiovascular diseases, stroke, and above average cases of death. This disease can be more complicated and worrisome among children and adolescents compared to adults, as it is proven to be an invasive disease with a high incidence of treatment failure and early side effects [8]. Therefore, since there is a relationship between the risk effects of diabetes and the duration of the disease, quick diagnosis and proper treatment are of the essence [9]. As a result, in addition to proper management, prevention of child obesity and type-2 diabetes should be high on the agenda in the healthcare system. Otherwise, the next generation might live a shorter life than their parents [10]. It is worth noting that sociocultural, geographical as well as environmental factors play a role in developing type-2 diabetes [11].

Early prevention – particularly targeting adolescents – can be a key measure to prevent diabetes [12]. Adolescence and school years constitute a critical stage of life to provide future health and harmony. In fact, there is a great potential to increase the health condition of this age group [13]. In this period, adolescents upgrade their skills and gradually assume more responsibility for their health [14].

Today adolescents are leading an unhealthy lifestyle. They tend to eat fast food, fatty food, and unhealthy food. In addition, they are averse to exercising. These are the leading factors contributing to type-2 diabetes [12]. Unhealthy behavior leading to type-2 diabetes is also potentially connected to other aspects such as awareness of and attitude toward the disease [12]. An individual’s attitude is defined as one’s tendency to show learned positive or negative reaction to an object, situation, concept, or a particular person [15].

Positive and negative attitudes can affect the course of a chronic disease [16]. A negative attitude toward a disease is typically regarded as a risk factor leading to psychological and emotional problems [17]. Adopting healthy behavior and measures might depend on the person’s attitude toward the risk. factor, that is, the perceived susceptibility – one’s belief and perception of the potential risk – and the perceived severity – one’s perception of the side effects and complications of the disease [18].

There is no precise instrument to assess adolescents’ health beliefs regarding type-2 diabetes. Studies that have attempted to design instruments mainly focus on diabetics’ quality of life (DQOL) [19], diabetic self-care [20], diabetic knowledge [21], and diabetics’ awareness, attitude and behavior [22]. This study aimed at designing a reliable and valid instrument to assess adolescents’ health beliefs regarding type-2 diabetes to be used in education and prevention programs in the future.

## Methods

### Design and setting

This research is a methodological investigation following a multistage sampling carried out on 770 adolescents and performed in Tehran.

### Inclusion and exclusion criteria

The inclusion criteria for research were included seventh, eighth, and ninth grade students, voluntary and informed consent, and not having type 1 or 2 diabetes. Also, the exclusion criteria from the study was the incomplete questionnaire.

### Participants

Seven hundred seventy male and female adolescents (between 13 and 15) from Tehran participated in this project. The population was selected through a multistage sampling. Tehran was divided into five sections: northeast, southeast, center, northwest, and southwest. From each section, one area was selected. Then, a girl’s school and a boy’s school were randomly selected from each area – totally 10 schools. Finally, 26 eligible students were randomly selected from each grade in each school.

### Designing the instrument

1. *Systematic review of literature and the relevant instruments*: In this step for the systematic review of literature and the relevant instruments electronic search was carried out from Persian databases of SID and Magiran and english databases of PubMed, Science Direct, Scopus, Web of science, and Google scholar was carried out. The keywords such as type 2 diabetes, diabetes mellitus, attitude, health beliefs, adolescents, teenagers, methodological, development, instrument, validity and reliability were used for searching. 30 questionnaires, 34 papers, and 10 theses were consulted to design the questionnaire.
2. *Determining and designing the items of the instrument through the existing documents, papers, and questionnaires in Iran and other countries*: questions were designed by extensive literature review and the related existing questionnaires. The pool of questions was created by 57 Items. By removing similar (23 items) or inappropriate sentences (8 items), a draft questionnaire with 26 questions was designed.
3. *Validity of the instrument*: Face, content and construct validity were used to determine the validity of the instrument.

#### Content validity

Both quantitative and qualitative methods were employed to determine content validity. In quantitative analysis of content validity, content validity ratio (CVR) as well as content validity index (CVI) were calculate [23]. To determine content validity ratio, 11 experts (7 specialists in health education & health promotion, 2 endocrinologists and 2 pediatric endocrinologists) were asked to evaluate each question and comment on their significance. The following formula was used to determine the content validity ratio:

$$\mathrm{CVR}=\frac{{\mathrm{n}}_{\mathrm{E}}-\raisebox{1ex}{$\mathrm{N}$}\!\left/ \!\raisebox{-1ex}{$2$}\right.}{\raisebox{1ex}{$N$}\!\left/ \!\raisebox{-1ex}{$2$}\right.}$$

Finally, the resulting CVR amounts higher than 0.59 were accepted based on Lawshe Table. In addition, modifications were made to the questionnaire after negotiations and discussion with the research team.

The CVI result was determined by calculating the sum of scores for each item – 3 and 4 (the highest score) – as well as the following formula. The resulting CVI amounts higher than 0.79 were accepted. In the qualitative analysis, the experts were asked to evaluate each item.

$$\mathrm{CVI}=\sum \frac{\mathrm{Number\ of\ answers }\ 3\mathrm{\ or }\ 4 }{\mathrm{ Total\ Number\ of\ answer}}$$

#### Face validity

Face validity was determined both qualitatively and quantitatively.

#### Qualitative face validity

During this stage, to determine the qualitative face validity of the questionnaire, 20 students of 13-15 years of age (10 boys and 10 girls), who had been selected through multistage sampling, were interviewed face to face. Their views on the questions were sought with regard to levels of difficulty, consistency, and ambiguity. Finally, the necessary modifications were made considering the target group feedback.

#### Quantitative face validity

The quantitative face validity of the questionnaire was employed to remove the inappropriate questions and determine the significance of each question. The same 20 students were asked to examine the questions based on a 5-point Likert scale and select one: Very important (5 points), important (4 points), rather important (3 points), a little important (2 points) and not important (1 point).

Then, the impact score of each question was calculated following this formula:

$$\mathrm{Impact\ score}=\mathrm{ Frequency }\ (\mathrm{\%}) \times \mathrm{ Importance}$$

The impact scores of higher than 1.5% were considered acceptable [24].

#### Construct validity

To determine construct validity, exploratory factor analysis with varimax rotation and confirmatory factor analysis were employed. The adequacy of samples to perform exploratory factor analysis was carried out by two tests of sampling adequacy Kaiser-Mayer-Olkin (KMO) and Bartlett’s test of sphericity (BT). Confirmatory factor analysis was investigated by Adjusted Goodness of Fit Index) AGFI, (Root Mean Square Error of Approximation) RMSEA, (Comparative Fit Index (CFI) and Goodness of Fit Index (GFI).

#### Reliability of the instrument

Cronbach’s alpha was calculated to determine the internal consistency, which is known as the internal index for the variables. Cronbach’s alpha of between 70 and 80% was set to be adequate and acceptable for internal consistency [25]. Test–retest was employed to investigate the stability of the instrument over time. The questionnaire was completed by 40 adolescents (20 boys and 20 girls) with an interval of 2 weeks.

**Fig. 1:**
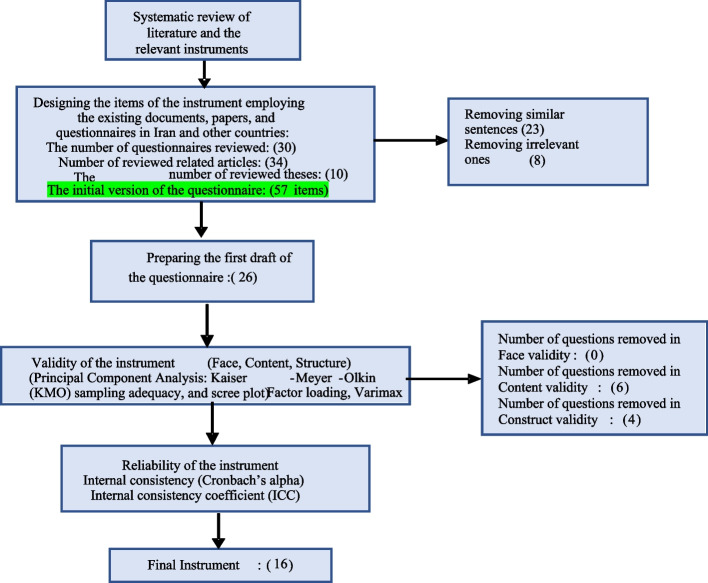
Flowchart of the design and psychometric stages of the questionnaire

A correlation coefficient of higher than 0.7 was considered acceptable for ICC (Internal Consistency Coefficient) (Fig. 1).

### Data analysis

CVR/CVI and impact scores were calculated to determine content and face validity respectively. Construct validity was calculated through exploratory factor analysis (EFA) with varimax rotation and confirmatory factor analysis (CFA). Cronbach’s alpha coefficient was calculated to determine the internal consistency. To check the stability of the instrument, the internal correlation coefficient (ICC) was calculated. SPSS16 and EQS6.1 were consulted for data analysis.

## Results

### The participants

The participants’ descriptive characteristics are listed in Table 1.

**Table 1:** Demographic information of the participants

**Variables**	**Sub Group**	**Number**	**Percent**
Age	13-year-old	267	35
	14-year-old	247	32
	15-year-old	256	33
Gender	Girl	453	59
	Boy	317	41
Grade of education	Seventh	270	36
	Eighth	242	31
	Ninth	258	33
Fathers’ occupation	Employee	223	29
	Self-employed	407	54
	Unemployed	48	6
	Retired	92	11
Mothers’ occupation	Employed	247	32
	House keeping	523	68
Fathers’ education	Illiterate	25	3
	Primary	58	7
	Intermediate	155	20
	Secondary	296	39
	Institutes/College	236	31
Mothers’ education	Illiterate	17	2
	Primary	72	9
	Intermediate	116	15
	Secondary	324	43
	Institutes/College	241	31
Economic situation	Poor	67	8
	Middle	268	35
	Good	304	39
	Excellent	131	17

### Designing the questions

Having explored various instruments and studied relevant papers and documents –questionnaires [23], papers [26], and theses [27] concerning type-2 diabetes – the research team came up with an initial list of 57 items. Then, similar sentences (23 items) and irrelevant ones (8 items) were removed, and the first draft of the questionnaire with 26 questions was designed.

### Content validity

At this stage, six items were removed and 20 questions remained. The range of I-CVI was from 1 to 0.79. The values of S-CVI/Ave and S-CVI/UA were respectively 0.94 and 0.70.

### Qualitative face validity

In the qualitative phase of face validity, based on participants’ feedback some questions were modified and no item was removed.

### Quantitative face validity

In the quantitative phase, the students’ responses were analyzed. Since the impact scores of all the questions were higher than 1.5, no question was removed at this stage. The questionnaire with 20 items was ready to be checked for construct validity.

### Construct validity

In this study 20 samples were considered for each item and whit design effect 1.9 and %1 missing, 770 sample was calculated. 770 eligible boy and girl students of 13-15 years were selected by multistage sampling for determining questionnaire construct validity (385 samples for exploratory factor analysis and 385 samples for confirmatory factor analysis). Before EFA and CFA the mean, standard deviation, skewness, kurtosis, celing and floor effect of Items and factors were performed. The result showed that the data have a normal distribution and the celing effect and the floor effect are insignificant (Table 2).

**Table 2:** The mean, standard deviation, skewness, kurtosis, celing and floor effect of perceived self-efficacy, behavioral beliefs, perceived severity, and perceived sensitivity

Factors	Mean	Std. Deviation	Skewness	Kurtosis	Floor effect	Celing effect
Perceived Self-efficacy	19.44	3.86	-.260	-.740	0.7	12.7
Behavioral Beliefs	17.67	3.64	.106	.057	0.5	6
Perceived Severity	14.29	3.20	-.283	.004	0.7	6
Perceived Sensitivity	6.55	2.02	-.090	-.752	2.2	8.1

The KMO index and Bartlett’s test of sphericity showed the adequacy of the data for performing factor analysis. The KMO test result demonstrated the adequacy of the data (KMO = 0/85), so did the Bartlett’s test (*p* < /001). In this study, four factors with noticeable value (Eigenvalues) of above 1 were extracted. Having determined the number of extractable factors and checking all rotation methods through varimax rotation, four main factors were extracted. The first factor (perceived self-efficacy) included five questions with the particular value of 2.76. The second factor included five questions with the particular value of 2.56. The third factor included four questions with the particular value of 2.07, and the fourth factor included two questions with the particular value of 1.31 (Table 3). Four items that loaded less than these amounts were removed from the questionnaire. Therefore, the questionnaire was reduced to 16 items.

**Table 3:** Factor load of health beliefs questionnaire items based on factor analysis with varimax rotation

**Rotated Component Matrix**				
	**Component**			
	1	2	3	4
I can do regular exercise to prevent type-2 diabetes.	0.75			
I am certain that I can prevent type-2 diabetes.	0.71			
I can avoid eating unhealthy food (fatty, salty, and sweet food) to prevent type-2 diabetes.	0.66			
I am certain that I can keep mentally sane to prevent type-2 diabetes.	0.66			
I can stop smoking to prevent type-2 diabetes.	0.60			
Viewing TV and using computer and tablet (above 3 h per day) can increase the risk of type-2 diabetes.		0.76		
Vitamins D and K as well as minerals play a vital role in controlling or preventing type-2 diabetes.		0.68		
Adequate sleep (7 to 8 h) decreases the risk of type-2 diabetes.		0.62		
Depression and stress can contribute to type-2 diabetes.		0.62		
Normal levels of cholesterol can prevent type-2 diabetes.		0.52		
Diabetes is a debilitating disease.			0.76	
Diabetes can reduce life span.			0.71	
Diabetes can restrict one’s occupational role and family responsibilities.			0.71	
Diabetes can affect the quality of life of the patient and his/her family.			0.61	
I don’t suffer from diabetes and don’t need blood sugar control.				0.80
Only overweight people contract diabetes.				0.74
% of Variance	28.60	10.08	8.26	7.25
Cumulative %	28.60	38.67	46.94	54.19

**Fig. 2:**
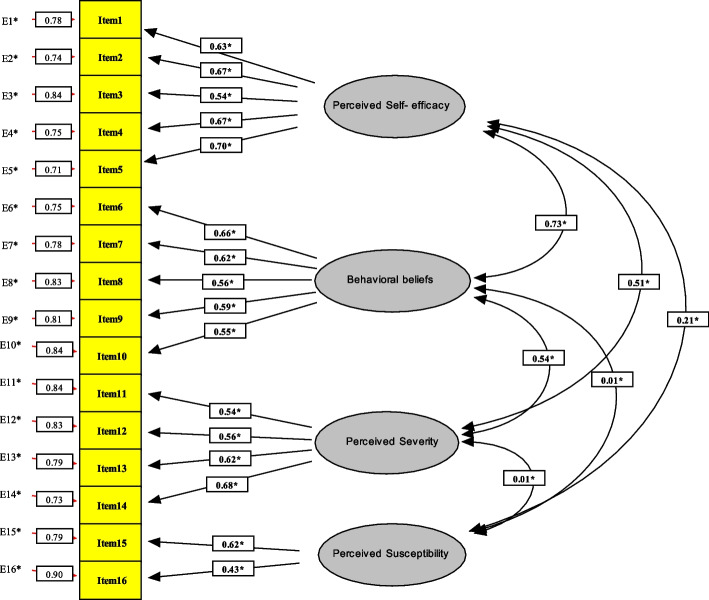
Diagram of confirmatory factor analysis

In the present study, the results of the confirmatory factor analysis as well as the following values were obtained (Fig. 2): Chi-Square to df = 3.96, RMSEA = 0.05, RMR = 0.06, AGFI = 0.91, GFI = 0.94, and CFI = 0.90.

And, according to Table 4, the results of confirmatory factor analysis were acceptable.

**Table 4:** Assess the fitness of the model

**Model fitness indicators**	**Indexes values**
Chi-Square	380.596
Degrees of Freedom (DF)	96
Root Mean Square Error of Approximation (RMSEA)	0.05
Root Mean-square Residual (RMR)	0.06
Adjusted Goodness of Fit Index (AGFI)	0.91
Goodness of Fit Index (GFI)	0.94
Comparative Fit Index (CFI)	0.90

### Reliability

The internal consistency of the instrument using Cronbach’s alpha coefficient turned out to be 78% for the whole instrument (16 questions), and above 0.70 in all four factors. The stability of the instrument using ICC (Intra class Correlation Coefficient) was all above 0.70 and fluctuated between 70 and 83% for the factors in the instrument. The reliability of test-retest stood at 0.73 for the whole instrument (Table 5).

**Table 5:** The intra-class correlation coefficient and the Cronbach’s α coefficient of questionnaire

**Factors**	**Number of Items**	**Cronbach’s alpha coefficient**	**ICC (** ***N*** ** = 40)**
Perceived Self-efficacy	5	0.70	0.70
Behavioral Beliefs	5	0.83	0.70
Perceived Severity	4	0.83	0.78
Perceived Susceptibility	2	0.70	0.87
Total	16	0.70	0.73

### The final instrument

The Final questionnaire was confirmed with 16 questions in four dimensions (based on the type and content of the questions): perceived self-efficacy, behavioral beliefs, perceived severity, and perceived susceptibility. The scoring of the items was in a 5-point Likert scale: strongly agree (5 points), agree (4 points), no idea (3 points), disagree (2 points), and strongly disagree (1 point). However, for two questions the reverse was applied: strongly agree (1 point), agree (2 points), no idea (3 points), disagree (4 points), and strongly disagree (5 points).

Finally, in order to measure the health beliefs of 770 adolescents, a questionnaire made with porslin (https://survey.porslin.ir/s/d1KMSO) was sent to the samples (due to the corona pandemic). Then the collected data was entered into spss16 and analyzed.

## Discussion

This study aimed at designing a psychometric instrument for assessing adolescents’ health beliefs regarding type-2 diabetes. Content validity, face validity, and construct validity were calculated to meet the scientific requirements of the research study. In this study based on Lawshe Table, the CVR above 0.0 and CVI above 0.79 were accepted. In the quantitative phase, the impact score of all the items was above 1.5; therefore, all the items were kept as proper for the next round of analysis. The results indicate that the questionnaire designed has been simple to understand and respond to. It also highlights the fact that the expressions used have been relevant and significant.

In this research, the KMO value for all the constructs was 0.85 and the significance level in the Bartlett’s test of sphericity (BT) was 0.001 indicating the adequacy of sampling for factor analysis. Confirmatory factor analysis was employed to determine construct validity. EQS6.1 showed AGFI to be 0.91 demonstrating an acceptable value of Model-Fit. In addition, RMSEA and the ratio of Chi-Square to df were at an acceptable level.

The construct validity of the instrument to assess health beliefs of adolescents regarding type-2 diabetes was obtained by exploratory factor analysis. This analysis gave rise to the extraction of four factors: perceived self-efficacy, behavioral beliefs, perceived severity, and perceived susceptibility. Perceived self-efficacy involves the individual’s certainty of one’s ability to organize activities and successful implementation until desired goals are achieved in particular situations. The higher the degree of certainty, the easier it is to adopt health measures [28].

To operationalize the construct of self-efficacy, the onus is on experts to exploit strategies such as verbal encouragement, creating models, emotional encouragement, practice, and accepting failure as an integral part of learning [29]. Considering the significance of self-efficacy in adopting preventive measures as well as complying with the treatment procedure, health experts and policymakers need to pay particular attention in its promotion to prevent chronic diseases such as type-2 diabetes.

The construct of behavioral beliefs was the second extracted factor in this study. Behavioral beliefs is defined as an individual’s belief in the fact that following a particular behavior is normally accompanied with positive or negative features or particular consequences [30]. Therefore, a person who has firm beliefs in the positive consequences of one’s action, he/she will also have a positive attitude toward that behavior. On the contrary, someone who firmly believes in the negative results of an action, he/she will take a negative attitude toward that behavior [31]. What is expected from this discussion is that educational interventions against type-2 diabetes among adolescents can control or stop the growth of this disease by promoting behavioral beliefs as well as helping adolescents adopt a positive attitude toward a healthy lifestyle.

Two other factors extracted by exploratory factor analysis were perceived Susceptibility and perceived severity. Perceived sensitivity, in fact, refers to a person’s belief regarding the possibility of contracting a disease as a result of following a particular behavior. On the other hand, perceived severity has more to do with a person’s belief regarding the potential range of injuries caused by a disease or the harmful effects of a particular behavior [32]. As type-2 diabetes can be more complicated and worrisome among children and adolescents compared to adults [33], high levels of perceived severity can help individuals adopt and commit themselves to healthy measures and behaviors [34]. If education and training help children and adolescents show more Susceptibility to diseases, perceive their severity and follow the right course of action, they are highly likely to pursue the right pattern of behave [35]. According to general consensus reached in international conventions, intervention programs concerning prevention of chronic diseases should begin in childhood and particularly in schools [36]. Health education in schools can promote the culture of health in any society [37]. Therefore, the significance of perceived Susceptibility and perceived severity needs to be taken into consideration in the design of preventive interventions against type-2 diabetes among adolescents.

In this study, Cronbach’s Alpha Coefficient was used to measure internal consistency reliability. Representing the degree of consistency among a group of items measuring a construct. The alpha value should be at least 0.7 or beyond so that a question can be retained in an instrument [38]. In addition, test-retest – the most valid measure of intraclass correlation coefficient – was used to determine the consistency of the instrument. In the present study, the result obtained from the reliability of the instrument showed that the Cronbach’s alpha coefficient stood at 0.78, and the consistency coefficient of 0.73 for each factor represents the internal consistency of the attitude questionnaire concerning type-2 diabetes among adolescents.

## Conclusion

The results of this study provide strong evidence concerning the strength of factor structure and the statistical reliability of the instrument that assesses health beliefs about type-2 diabetes. Relying on validity and reliability has proven to be crucial in studies particularly for the purpose of designing, implementing, and evaluating interventions against critical issues such as type-2 diabetes. These methods of analysis provide valuable information for planners and policymakers.

The distribution of the sample in the population in terms of number provides the generalizability of the data, making it one of the strengths of this research. In addition, the instrument designed has an acceptable level of validity and reliability to assess health beliefs of adolescents regarding type-2 diabetes. One of the limitations of the study is the method of self-report used in completing the questionnaire.

Understanding the impact of adolescents’ health beliefs about type-2 diabetes is critical in prevention, and such knowledge should inform the education and training that adolescents and parents receive.

## Data Availability

All data relevant to the study are included in the article.
